# Prevalence of plasma small dense LDL is increased in obesity in a Thai population

**DOI:** 10.1186/s12944-015-0034-1

**Published:** 2015-04-18

**Authors:** Sirikul Kulanuwat, Rungsunn Tungtrongchitr, David Billington, Ian G Davies

**Affiliations:** Department of Tropical Nutrition & Food Science, Faculty of Tropical Medicine, Mahidol University, Bangkok, Thailand; School of Pharmacy and Biomolecular Sciences, Liverpool John Moores University, Byrom Street, Liverpool, L3 3AF UK; Faculty of Education, Health and Community, Liverpool John Moores University, Barkhill Road, Liverpool, L17 6BD UK

**Keywords:** BMI, Iodixanol, Small dense LDL, Large buoyant LDL, Obesity, Triglyceride, Ultracentrifugation

## Abstract

**Background:**

Plasma low density lipoprotein (LDL) particles vary in size, density, electrical charge and chemical composition. An increased presence of small dense LDL (sdLDL), along with raised triglyceride concentrations and decreased high density lipoprotein (HDL) cholesterol concentrations is commonly known as the atherogenic triad and has been observed in some cases of obesity, principally in Europe and America. This study examines the prevalence of sdLDL in the plasma of an obese (BMI ≥ 25 kg/m^2^) Thai population.

**Methods:**

Plasma from fasted obese (n = 48) and non-obese (n = 16) Thai participants was subjected to density gradient ultracentrifugation in iodixanol to separate lipoproteins. Gradients were unloaded top-to-bottom into 20 fractions which were assayed for cholesterol, triglyceride, apo B and apo A-1 to identify lipoprotein types and subtypes.

**Results:**

LDL cholesterol was subfractionated into LDL I + II (fractions 3–6, ρ = 1.021-1.033 g/ml) which was considered to represent large buoyant LDL (lbLDL), LDL III (fractions 7–9, ρ = 1.036-1.039 g/ml) which was considered to represent sdLDL, and, LDL IV (fractions 10–12, ρ = 1.044-1.051 g/ml) which was considered to represent very sdLDL. Concentrations of LDL III and IV were increased by 15-20% in obese participants whilst that of LDL I + II was concomitantly decreased by 10%. This was accompanied by a 50% increase in plasma triglyceride concentrations and 15% decrease in HDL cholesterol concentrations. Only 3/16 (19%) non-obese participants had a pattern B LDL cholesterol profile (peak density of >1.033 g/ml), whilst 28/48 (58%) obese participants were pattern B. When expressed as a fraction of the LDL concentration, total sdLDL (i.e. LDL III + IV) showed highly significant correlations to plasma triglyceride concentrations and the triglyceride/HDL cholesterol ratio.

**Conclusions:**

The prevalence of sdLDL is increased in obesity in a Thai population such that they demonstrate a similar atherogenic triad to that previously observed in European and American populations.

## Background

Obesity is an increasing worldwide health concern, and a known risk factor for several chronic diseases including type-2 diabetes, cardiovascular disease, skeletal disorders and some cancers [1-5]. Whilst a body mass index (BMI) of ≥30 kg/m^2^ is generally accepted to define obesity in Europe and North America, a lower threshold of 25 kg/m^2^ is more appropriate to define obesity in Thai and south-east Asian populations [6-9]. Indeed, a recent study has proposed a BMI cut-off of 23 or 24 kg/m^2^ for Thai men and women respectively to predict at least one cardiovascular risk factor [10].

LDL consists of a continuum of particles varying in size, density, electrical charge, and chemical (lipid and apo-protein) composition. Density gradient ultracentrifugation in high concentrations of salt (usually KBr) was originally used to identify and quantitate LDL subclasses in plasma. Using this method, four LDL subclasses were identified in the density range 1.019-1.060 g/ml; namely LDL-I (large buoyant, 1.019-1.023 g/ml), LDL-II (intermediate, 1.024-1.034 g/ml), LDL-III (small dense, 1.034-1.044 g/ml) and LDL-IV (very small dense, 1.044-1.060 g/ml) [11,12]. Krauss and colleagues have simplified this to define two LDL phenotypes; so-called pattern A individuals who are characterized by a predominance of large buoyant LDL (lbLDL) in plasma whilst pattern B individuals have a predominance of small dense LDL (sdLDL) [12,13].

Some studies have observed an increased presence of sdLDL particles in the plasma of obese participants [14,15]. This is associated with raised plasma triglyceride concentrations and decreased HDL-cholesterol concentrations, forming the so-called ‘atherogenic lipid triad’ [16]. sdLDL is more atherogenic partly because it has a lower affinity for the LDL receptor and hence remains in the circulation longer, but also because its lipid and protein components are more prone to chemical modification (e.g. oxidation or glycation) and uptake by scavenger receptors [17]. Indeed, it has been estimated that participants with raised sdLDL concentrations have an approximate 3- to 7-fold increase in risk of developing coronary heart disease [18].

Whilst an increased prevalence of sdLDL has been associated with obesity in a variety of populations, this has not been investigated in Thai populations, and this was the primary aim of this study. Density gradient ultracentrifugation in iodixanol was used as this allowed the identification and quantitation of all LDL subclasses, not just sdLDL. Iodixanol is an iso-osmotic, iodinated compound (M_r_ 1550) which has been used to separate LDL subclasses by density gradient ultracentrifugation [19,20]. It has the advantage over high concentrations of KBr in that density gradients can be generated during a centrifuge run and, when carried out in a vertical or near-vertical rotor, results in much shorter centrifuge run times of 2–3 hours.

## Results

Of the 64 Thai participants recruited to the study, 48 (75%) had a BMI ≥ 25 kg/m^2^ and hence were classed as obese (Table 1). Both obese and non-obese participants had similar ages and plasma total cholesterol concentrations were similar in both groups whilst plasma triglyceride concentrations were significantly increased by approximately 50% in obese compared to non-obese participants (Table 1).

**Table 1:** **Characteristics of plasma lipids in obese and non-obese Thai participants**

	**Non-obese**	**Obese**	***p*** **value**	**Adjusted** ***p*** **value**
	**(BMI < 25 kg/m** ^**2**^ **)**	**(BMI ≥25 kg/m** ^**2**^ **)**		
n (%)	16 (25.0)	48 (75.0)		
Sex (M/F)	8/8	12/36		
Age (year)	46.3 ± 12.6	46.4 ± 11.4	0.980	-
Total cholesterol (mmol/L)	5.18 ± 1.03	5.24 ± 0.99	0.833	-
Triglyceride (mmol/L)	1.04 ± 0.55	1.64 ± 0.59	0.001	0.002
VLDL-C (mmol/L)	0.30 ± 0.12	0.42 ± 0.14	0.004	0.010
LDL-C (mmol/L)	2.90 ± 0.89	3.03 ± 0.82	0.612	-
LDL I + II (mmol/L)	1.56 ± 0.41	1.37 ± 0.40	0.116	-
LDL III (mmol/L)	1.05 ± 0.43	1.28 ± 0.41	0.061	-
LDL IV (mmol/L)	0.27 (0.19-0.31)	0.34 (0.27-0.46)	0.003	0.035
LDL I + II /LDL	0.55 ± 0.06	0.46 ± 0.07	<0.001	0.002
LDL III /LDL	0.36 ± 0.05	0.42 ± 0.05	<0.001	0.002
LDL IV /LDL	0.09 ± 0.02	0.12 ± 0.03	<0.001	0.003
HDL-C (mmol/L)	1.43 ± 0.25	1.21 ± 0.22	0.001	0.004
Apo-A1 (g/L)	1.42 ± 0.27	1.22 ± 0.25	0.010	0.014
Apo-B (g/L)	0.86 ± 0.24	0.92 ± 0.25	0.390	-

Data are n (%) or mean ± SD or median (interquartile range). BMI, body mass index; VLDL-C, very low-density lipoprotein cholesterol; LDL-C, low-density lipoprotein cholesterol; LDL I + II, low-density lipoprotein subclasses I and II; LDL III, low-density lipoprotein subclass III; LDL IV, low-density lipoprotein subclass IV; HDL-C, high-density lipoprotein cholesterol. Means were compared between non-obese and obese groups by Independent-Sample T test, except LDL IV when *p* values were calculated from Mann–Whitney U test. P-values were adjusted for age and gender by Binary Logistic Regression.

**Figure 1:**
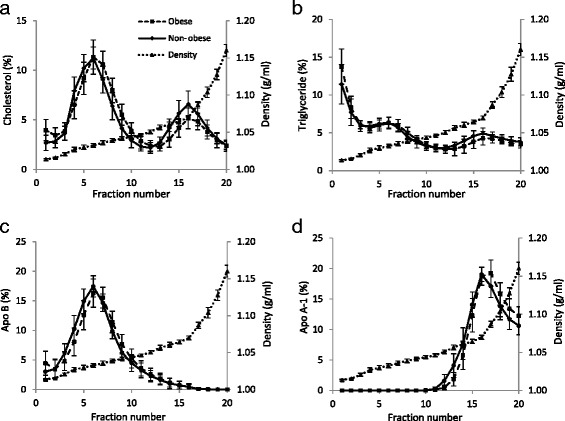
Lipid profiles of iodixanol density gradients. Plasma gradient fractions, from obese and non-obese Thai participants, were analysed for **(a)** Cholesterol, **(b)** triglyceride **(c)** apo-B and **(d)** apo-A1. Values are means ± SD of 16 non-obese participants (indicated by ●) and 48 obese participants (indicated by ■). Gradient densities are means ± SD of all 64 gradients (indicated by ▲).

Ultracentrifugation of plasma in self-generating gradients of iodixanol allowed separation of lipoproteins into their constituent types and subtypes. Under the conditions used in this study, LDL was identified as a broad peak of cholesterol (Figure 1a) and triglyceride (Figure 1b) between fractions 3–12 at a density of 1.021-1.051 g/ml. Apo-B, the principal apo-protein associated with LDL, showed exactly the same profile in fractions 3–12 of the gradients (Figure 1c). A second broad peak of cholesterol (Figure 1a) and triglyceride (Figure 1b) between fractions 13–20 was identified as HDL and occurred at density 1.057-1.161 g/ml (Figure 1a); this was confirmed by apo-A1 having a similar distribution profile (Figure 1d). Participants fasted before venipuncture, when chylomicrons would be expected to be absent. Thus, the large amount of triglyceride (Figure 1b) and smaller amount of cholesterol (Figure 1a) present in fractions 1–2 was interpreted as VLDL since this lipoprotein is known to carry mainly triglyceride.

Recovery of cholesterol, triglyceride, apo-B and apo-A1, expressed as a percentage of that loaded onto the gradients, was 100.1 ± 4.0%, 90.9 ± 7.7%, 90.6 ± 7.2% and 88.9 ± 6.4% respectively (mean ± SD of 64 observations). Plasma VLDL-, LDL- and HDL-cholesterol concentrations were calculated by summing recoveries in fractions 1–2, 3–12 and 13–20 respectively. Plasma HDL-cholesterol was significantly decreased by 15% in obese participants compared to non-obese participants, VLDL-cholesterol was increased by 40% whilst LDL-cholesterol was unchanged (Table 1). Concentrations of plasma HDL- and LDL-cholesterol derived from the density gradients showed excellent agreement with those determined by selective precipitation and the Friedewald equation (data not shown).

LDL cholesterol was further fractionated into:

- LDL I + II (fractions 3–6, ρ = 1.021-1.033 g/ml) which were considered to represent large buoyant and intermediate density LDL,
- LDL III (fractions 7–9, ρ1.036-1.039 g/ml) which was considered to represent sdLDL, and,
- LDL IV (fractions 10–12, ρ = 1.044-1.051 g/ml) which was considered to represent very sdLDL.

Whilst plasma total cholesterol concentrations were similar in obese and non-obese participants, cholesterol concentrations of the most dense LDL subclass, namely LDL IV, were significantly increased by approximately 20% in obese participants (Table 1). Similarly, the cholesterol concentration of LDL III was increased by approximately 15% whilst that of the larger, more buoyant LDL I + II was decreased by approximately 10%, although both of these changes did not reach statistical significance (Table 1). Thus, there was a marked shift towards LDL of higher density in obese Thai participants and this was further evidenced in three ways. Firstly, when expressed as a fraction of total LDL, plasma concentrations of LDL I + II subclasses (hereinafter known simply as large buoyant (lb) LDL) were significantly lower in obese compared to non-obese participants whilst those of LDL III and LDL IV subclasses were significantly increased (Table 1). Secondly, close examination of the cholesterol and apo-B distribution profiles in fractions 3–12 of Figure 1a and c shows a marked shift towards a higher density, equivalent to approximately half of one fraction, in the plasma of obese participants. This effect was less apparent, although still noticeable, in the triglyceride profile (Figure 1b). Finally, the density of the cholesterol peak following density gradient centrifugation in iodixanol has been used by Davies et al. [20] to identify so-called pattern A (ρ < 1.033 g/ml) and pattern B (ρ ≥ 1.033 g/ml) individuals. When this was applied to the participants studied here, only 3/16 (19%) of the non-obese participants had a pattern B lipid profile whilst 28/48 (58%) of the obese participants were pattern B; this difference was highly significant (Table 2).

**Table 2:** **Peak density of LDL cholesterol in obese and non-obese participants**

**LDL peak density (g/ml)**	**Non-obese (%)**	**Obese (%)**	**Total (%)**	**Chi-square**
				***p*** **value**
ρ < 1.033; Pattern A	13 (81.2)	20 (41.7)	33 (51.6)	0.009
ρ ≥ 1.033; Pattern B	3 (18.8)	28 (58.3)	31 (48.4)	
Total (%)	16 (100)	48 (100)	64 (100)	

Interestingly, analysis of the lipid profiles of the pattern A and B participants identified in this study showed that plasma triglyceride and total cholesterol concentrations were increased by approximately 40% and 15% respectively in pattern B participants (Table 3). Plasma VLDL- and LDL-cholesterol (plus apo-B) concentrations were increased in pattern B participants by 50% and 20% respectively, whilst HDL-cholesterol (and apo-A1) concentrations were similar in pattern A and B participants (Table 3).

**Table 3:** **Plasma lipids in pattern A and B participants**

	**Pattern A**	**Pattern B**	***p*** **value**	**Adjusted** ***p*** **value**
	**d < 1.033**	**d ≥ 1.033**		
	**(g/ml)**	**(g/ml)**		
Sex (M/F)	11/22	9/22	0.711	-
Cholesterol (mmol/L)	4.85 ± 0.72	5.62 ± 1.09	0.001	0.007
Triglyceride (mmol/L)	1.08 ± 0.35	1.93 ± 0.56	<0.001	<0.001
VLDL-C (mmol/L)	0.31 ± 0.11	0.48 ± 0.13	<0.001	<0.001
LDL-C (mmol/L)	2.73 ± 0.68	3.27 ± 0.90	0.009	0.016
HDL-C (mmol/L)	1.29 ± 0.27	1.23 ± 0.22	0.375	-
Apo-A1 (g/L)	1.28 ± 0.30	1.26 ± 0.24	0.758	-
Apo-B (g/L)	0.83 ± 0.22	0.99 ± 0.25	0.011	0.016

Data are means ± SD for pattern A participants (n = 33) and pattern B participants (n = 31). Means were compared between pattern A and pattern B groups by independent-Sample T tests. P-values were adjusted for age, gender and BMI by Binary Logistic Regression.

**Figure 2:**
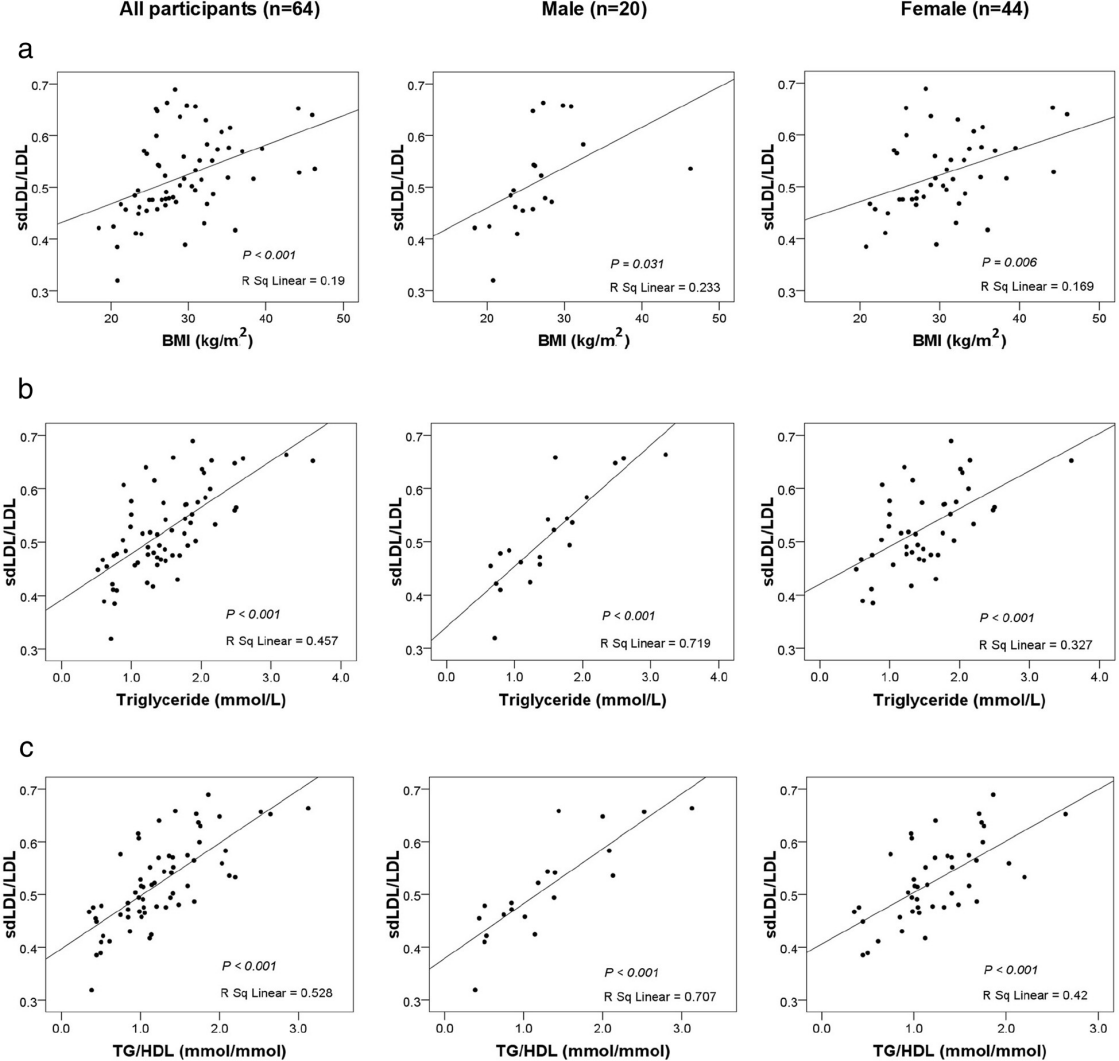
Correlation of sdLDL/total LDL ratio with **(a)** BMI, **(b)** plasma triglyceride, and, **(c)** plasma triglyceride (TG)/HDL-cholesterol ratio for all participants. n = 64, males (n = 20) and females (n = 44). Small dense LDL was defined as LDL III + IV. Correlation p-values and r^2^ values are given in each panel.

Finally, when expressed as a fraction of the LDL concentration, total sdLDL-cholesterol (i.e. LDL III + IV) showed highly significant positive correlations to BMI, plasma triglyceride concentrations and the triglyceride/HDL-cholesterol ratio (Figure 2). As judged by r^2^ values, the most significant correlations were with plasma triglyceride concentrations and the triglyceride/HDL-cholesterol ratio, and in all cases, correlations were stronger in males than females (Figure 2).

## Discussion

The ‘gold standard’ methods for studying LDL heterogeneity are density gradient ultracentrifugation and gradient gel electrophoresis which separate LDL subclasses on the basis of density and size respectively [21]. Depending upon the detailed methodology, 3–7 LDL subclasses have been identified by these techniques. Other methods which rely on different principles include nuclear magnetic resonance, ion mobility analysis, dynamic light scattering and homogenous assays (see [21]). Such varied methods, and the inevitable heterogeneity within separated LDL subclasses, makes comparison of results from different studies extremely difficult. We chose iodixanol density gradient centrifugation, not only because it separates LDL subclasses on the basis of density, but because iodixanol gradients are iso-osmotic. This maintains LDL particles in their native state compared to the inevitable osmotic damage that would be expected to occur in traditional high salt density gradients [20,22]. As a result, the density of sdLDL reported in this study (LDL III + IV, fractions 7–12 of Figure 1, ρ = 1.036-1.051 g/ml) is lower than that reported from KBr gradients (1.034-1.060 g/ml) [12,18].

A homogeneous assay for sdLDL, based on prior precipitation of VLDL and lbLDL by heparin and Mg^2+^ and subsequent assay of the supernatant using reagents for the LDL-cholesterol homogeneous assay, was first described by Hirano et al. [23]. More recently, the precipitation step has been modified to provide a direct sdLDL-cholesterol assay suitable for use in large scale intervention studies [24,25]. Such studies have reported sdLDL-cholesterol to represent 20-35% of total LDL-cholesterol with absolute plasma concentrations in the range 25–75 mg/dl (0.65-1.95 mmol/L) [25-28]. The results presented here show that sdLDL-cholesterol represents a somewhat higher proportion of total LDL-cholesterol; namely 45% for non-obese participants and 54% for obese participants (Table 1). However, the absolute plasma sdLDL-cholesterol concentrations of 1.32 and 1.62 mmol/L for non-obese and obese participants are within the range given above using the direct homogeneous assay. Interestingly, a recent study [29] defined sdLDL as cholesterol at density >1.040 g/ml (by equilibrium density gradient ultracentrifugation) as opposed to ≥1.036 g/ml used here; in this case, sdLDL-cholesterol still represented 30% of total LDL-cholesterol. Thus, despite the difficulty of comparing results from different studies, we are confident that the values for plasma sdLDL-cholesterol concentrations reported here are comparable to those reported elsewhere.

It was originally thought that sdLDL was formed in the circulation simply by delipidation of lbLDL [30]. However, there is now convincing evidence that sdLDL is formed directly from precursor lipoproteins secreted by the liver. Under conditions of high triglyceride availability (as occurs in obesity), the liver secretes greater amounts of large precursor lipoproteins which are delipidated by lipoprotein lipase and hepatic lipase directly to sdLDL [13,17]. As a result, plasma sdLDL concentrations are invariably raised in obesity and to date, most studies reporting elevated sdLDL concentrations in obesity have utilized either European or American populations (see, for example, [14,15,31,32]). However, a recent study from Korea has reported sdLDL to be elevated in obese participants, particularly those with large amounts of abdominal visceral fat [33]. To our knowledge, this is the first study to show that plasma concentrations of sdLDL-cholesterol are raised in an obese Thai population (Figure 1, Tables 1 and 2) and that, concurrent with this, concentrations of lbLDL-cholesterol were correspondingly decreased. Given that these obese participants had raised plasma triglyceride concentrations and decreased HDL-cholesterol concentrations (the atherogenic triad), it is to be expected that their risk of coronary heart disease would be increased considerably. It is noteworthy that the Thai National Health Examination Survey IV (carried out in 2009) reported the prevalence in the entire population of two components of the atherogenic triad, namely low HDL-cholesterol (<40/50 mg/dl, male/female) and high triglyceride (>150 mg/dl) to be 47.1% and 38.6% respectively [34].

An alternative way of analyzing the LDL density data from this study is to divide participants into pattern A and pattern B individuals based upon the peak density of their LDL-cholesterol [20]. Pattern B individuals, having a LDL-cholesterol peak density of ≥1.033 g/ml, were much more likely to be obese (Table 2), and had higher plasma concentrations of total cholesterol, triglyceride, VLDL-cholesterol, LDL-cholesterol and apo-B compared to pattern A individuals (Table 3). Surprisingly, pattern A and B individuals had similar plasma concentrations of HDL-cholesterol and apo-A1 (Table 3); these analytes would be expected to be decreased in pattern B individuals. Given that females are well known to have higher plasma HDL-cholesterol concentrations than males [35], one possible explanation for the unexpectedly high HDL-cholesterol concentration is that the pattern B population contained more females. However, this was not the case and the distribution of males and females was similar in the pattern A and B populations (Table 3). Although not measured in the present study, it is possible that dietary patterns are responsible for the unexpectedly high HDL-cholesterol concentrations in the pattern B population. For example, populations with a ‘meat and fast food’ dietary pattern in Korea show similar lipid patterns to the present study, with high plasma triglyceride concentrations and a decreased prevalence of low HDL-cholesterol concentrations [36]. In addition, the prevalence of low HDL-cholesterol has been reported to be significantly lower in urban areas of Thailand compared to rural areas, presumably due to different dietary patterns [34].

The association of obesity in a Thai population with the atherogenic triad is emphasized by the highly significant correlations of the sdLDL/LDL ratio with BMI, plasma triglyceride concentrations and the triglyceride/HDL-cholesterol ratio (Figure 2). The triglyceride/HDL-cholesterol ratio is a recently-reported, highly sensitive, surrogate marker for predicting cardiovascular events (and insulin resistance) in a variety of metabolic abnormalities [37-39].

## Conclusion

The prevalence of sdLDL in plasma is increased in obesity in a Thai population such that they demonstrate a similar atherogenic triad to that previously observed in European and American populations. It is to be expected that this would contribute significantly to an increased risk of coronary heart disease.

## Methods

### Study participants

Plasma samples were derived from a previous study investigating the occurrence of genetic variants in obese Thai participants from the Bangkok area [40]. All studied participants had been informed in writing of the intended use of their sample and provided written consent. The intervention studies were approved by the ethics committee of the Faculty of Tropical Medicine, Mahidol University REC in accordance with the declaration of Helskinki; reference number MUTM 2012-030-03. Blood was obtained from participants ≥20 years old after a 12-hour fast and taken into either acid-citrate-dextrose (ACD) or EDTA as anticoagulant. Plasma was obtained by centrifugation and stored at −80°C for up to 1 year before analysis. Body weight was measured using scales (ZEPPER TCS -150 L, China) and height was measured using a vertical-measuring rod (Microtoise, Poissy, France); BMI was calculated as weight (in kg) divided by height (in m) squared. Participants who had a BMI ≥25 kg/m^2^ were classified as obese whilst those with a BMI <25 kg/m^2^ were assumed to be non-obese [7].

### Iodixanol density gradient ultracentrifugation

Density gradients were prepared essentially as described by Davies et al. [20] using Optiprep™ (Axis-Shied PoC AS, Oslo, Norway) which is a commercially available solution of 60% (w/v) iodixanol and has a density of 1.32 g/ml. Plasma was adjusted to 12% (w/v) iodixanol by adding 1.6 ml of plasma (0.8 ml of ACD plasma + 0.8 ml of EDTA plasma) to 0.4 ml of Optiprep™ to give a final density of 1.064 g/ml. A 9% (w/v) iodixanol solution was prepared by adding 3.0 ml of Optiprep™ to 17 ml of phosphate –buffered saline (PBS) to give a final density of 1.048 g/ml. In order to generate the gradient, 3.4 ml of the 9% (w/v) iodixanol/PBS solution was transferred to a 4.9 ml Beckman Optiseal™ centrifuge tube. Using a cannula and syringe, 1.5 ml of the plasma sample made to 12% (w/v) with iodixanol was under-layered and the tube left for 30 min. Tubes were then centrifuged at 342,000 g at 16°C (deceleration program 5) for 2.5 h in an NVT65.2 near-vertical rotor in a Beckmann L8 ultracentrifuge. Previous work has shown that storage of plasma samples for up to 1 year, or mixing of ACD- and EDTA-plasma samples, had no effect upon the fractionation of lipoproteins by iodixanol density gradient untracentrifugation (data not shown).

Gradients were unloaded from top-to-bottom at a flow rate of 1 ml/min using a Labconco (Kansas, USA) Auto Densi-flow density gradient fractionator and harvested as 20 fractions using a Gilson (Luton, UK) FC205 fraction collector. The refractive index of gradient fractions was measured using an Abbe 5 refractometer (Bellingham and Stanley, Basingstoke, UK) and their density calculated by reference to the refractive indices of appropriate iodixanol density standards.

### Laboratory measurements

The cholesterol, triglyceride, apo-A1, and apo-B concentrations of plasma samples and gradient fractions were measured using diagnostic kits (and appropriate calibrators and serum quality controls) supplied by Randox Laboratories Ltd (Co. Antrim, UK). Analyses were carried out in a SPACE automated analyzer (Schiapparelli Biosystems, Inc., ENI Diagnostics Division, USA).

### Statistical analyses

The independent sample T-test was used to test the mean difference of plasma lipids and apo-protein concentrations between obese and non-obese groups, as well as between LDL-cholesterol peak densities. Data were tested for normality using the Kolmogorov**–**Smirnov test and for data that showed a non-normal distribution, the non-parametric Mann–Whitney U test was used. Chi-square analysis was used to analyze the association between LDL-cholesterol peak density and obesity status. Logistic regression was used to adjust the data for age, gender and BMI. Pearson correlation was used to analyze the correlation between two parameters. In all cases, a P-value of <0.05 was considered statistically significant and all tests were performed by SPSS program version 21.

## Abbreviations

BMI: Body mass index
lbLDL: Large buoyant LDL
sdLDL: Small dense LDL

## References

[1] Tchernof A, Despres JP (2013). Pathophysiology of human visceral obesity: an update. Physiol Rev..

[2] Vazquez G, Duval S, Jacobs DR, Silventoinen K (2007). Comparison of body mass index, waist circumference, and waist/hip ratio in predicting incident diabetes: a meta-analysis. Epidemiol Rev..

[3] Kawai M, de Paula FJ, Rosen CJ (2012). New insights into osteoporosis: the bone-fat connection. J Intern Med..

[4] Despres JP (2012). Body fat distribution and risk of cardiovascular disease: an update. Circulation..

[5] Strong AL, Burow ME, Gimble ME, Bunnell BA (2015). The obesity cancer paradigm: exploration of the interactions and cross-talk with adipose stem cells. Stem Cells..

[6] Kanazawa M, Yoshiike N, Osaka T, Numba Y, Zimmet P, Inoue S (2002). Criteria and classification of obesity in Japan and Asia-Oceania. Asia Pac J Clin Nutr..

[7] Thaikruea L, Seetamanotch W, Seetamanotch S (2006). Appropriate cut-off level of BMI for screening in Thai adults. J Med Assoc Thai..

[8] Peermsuwan U, Guntawongwan K, Buddhawongsa P (2008). Handling time in economic evaluation studies. J Med Assoc Thai..

[9] Pitayatienanan P, Butchon R, Yothasamut J, Aekplakorn W, Teerawattananon Y, Suksomboon N (2014). Economic costs of obesity in Thailand: a retrospective cost-of-illness study. BMC Health Serv Res..

[10] Samsen M, Hanchaiphiboolkul S, Puthkhao P, Tantirittisak T, Towanabut S (2012). Appropriate body mass index and waist circumference cutoffs for middle and older age group in Thailand: data of 19,621 participants from Thai epidemiologic stroke (TES) study. J Med Assoc Thai..

[11] Krauss RM (1991). Low density lipoprotein subclasses and risk of coronary artery disease. Curr Opin Lipidol..

[12] Krauss RM, Blanche PJ (1992). Detection and quantitation of LDL subfractions. Curr Opin Lipidol..

[13] Berneis KK, Krauss RM (2002). Metabolic origins and clinical significance of LDL heterogeneity. J Lipid Res..

[14] Magkos F, Mohammed BS, Mittendorfer B (2008). Effect of obesity on the plasma lipoprotein subclass profile in normoglycemic and normolipidemic men and women. Int J Obes (Lond)..

[15] Nikolic D, Katsiki N, Montalto G, Isenovic ER, Mikhailidis DP, Rizzo M (2013). Lipoprotein subfractions in metabolic syndrome and obesity: clinical significance and therapeutic approaches. Nutrients..

[16] Rizzoo M, Kotur-Stevuljevic J, Berneis K, Spinas G, Rini GB, Jelic-Ivanovic Z (2009). Atherogenic dyslipidemia and oxidative stress: a new look. Transl Res..

[17] Diffenderler MR, Schaefer EJ (2014). The composition and metabolism of large and small LDL. Curr Opin Lipidol..

[18] Mikhailidis DP, Elisaf M, Rizzo M, Bernies K, Griffin B, Zambon A (2011). “European panel on low density lipoprotein (LDL) subclasses”: a statement on the pathophysiology, atherogenicity and clinical significance of LDL subclasses. Curr Vasc Pharmacol..

[19] Sawle A, Higgins MK, Olivant MP, Higgins JA (2002). A rapid single-step centrifugation method for determination of HDL, LDL, and VLDL cholesterol, and TG, and identification of predominant LDL subclass. J Lipid Res..

[20] Davies IG, Graham JM, Griffin BA (2003). Rapid separation of LDL subclasses by iodixanol gradient ultracentrifugation. Clin Chem..

[21] Hirayama S, Miida T (2012). Small sense LDL: an emerging risk factor for cardiovascular disease. Clin Chim Acta..

[22] Yee MS, Pavitt DV, Tan T, Venkatesan S, Godsland IF, Richmond W (2008). Lipoprotein separation in a novel iodixanol density gradient, for composition, density, and phenotype analysis. J Lipid Res..

[23] Hirano T, Ito Y, Saegusa H, Yoshino G (2003). A novel and simple method for quantification of small dense LDL. J Lipid Res..

[24] Ito Y, Fujimura M, Ohta M, Hirano T (2011). Development of a homogeneous assay for measurement of small dense LDL cholesterol. Clin Chem..

[25] Fukushima Y, Hirayama S, Ueno T, Dohi T, Miyazaki T, Ohmura H (2011). Small dense LDL cholesterol is a robust therapeutic marker of statin treatment in patients with acute coronary syndrome and metabolic syndrome. Clin Chim Acta..

[26] Ai M, Otokozawa S, Asztalos BF, Nakajima K, Stein E, Jones PH (2008). Effects of maximal doses of atorvastatin versus rosuvastatin on small dense low-density-lipoprotein cholesterol levels. Am J Cardiol..

[27] Nozue T, Michishita I, Ito Y, Hirano T (2008). Effects of statin on small dense low-density-lipoprotein cholesterol and remnat-like particle cholesterol in heterozygous familial hypercholesterolemia. J Atherscler Thromb..

[28] Tokuna A, Hirano T, Hayashi T, Mori Y, Yamamoto T, Nagashima M (2007). The effects of statin and fibrate on lowering small dense LDL cholesterol in hyperlipidemic patients with Type 2 diabetes. J Atherscler Thromb..

[29] Winkler K, Jacob S, Mϋller-Schewe T, Hoffmann MM, Konrad T (2012). Ezetimibe alone and in combination lowers the concentration of small, dense low-density lipoproteins in type 2 diabetes mellitus. Atherosclerosis..

[30] Geiss HC, Bremer S, Barrett FHR, Otto C, Parhoefer KG (2004). In vivo metabolism of LDL subfractions in patients with heterozygous FH on statin therapy: rebound analysis of LDL subfractions after LDL apheresis. J Lipid Res..

[31] MacLean PS, Bower JF, Vadlamudi S, Green T, Barakat HA (2000). Lipoprotein subpopulation distributions in lean, obese and type 2 diabetic women: a comparison of African and white Americans. Obes Res..

[32] Bioletto S, Golay A, Munger R, Kalix B, James RW (2000). Acute hyperinsulinemia and very-low-density and low-density lipoprotein subfractions in obese subjects. Am J Clin Nutr..

[33] Kim S, Lee H, Lee DC, Lee HS, Lee JW (2014). Predominance of small dense LDL differentiates metabolically unhealthy from metaboloically healthy overweight adults in Korea. Metabolism..

[34] Aekplakorn W, Taneepanichskul S, Kessomboon P, Chongsuvivatwong V, Putwatana P, Sritara P (2014). Prevalence of dyslipidemia and management in the Thai Population, National Health Examination Survey IV, 2009. J. Lipids..

[35] Wilson PWF, Abbott RD, Castelli WP (1988). High density lipoprotein cholesterol and mortality. Arterioscler Thromb Vasc Biol..

[36] Shin HJ, Cho E, Lee HJ, Fung TT, Rimm E, Rosner B (2014). Instant noodle intake and dietary patterns are associated with distinct cardiometabolic risk factors in Korea. J Nutr..

[37] Fan X, Liu EY, Hoffman VP, Potts AJ, Sharma B, Henderson DC (2011). Triglyceride/high-density lipoprotein cholesterol ratio: a surrogate to predict insulin resistance and low-density lipoprotein cholesteral particle size in nondiabetic patients with schizophrenia. J Clin Psychiatry..

[38] Nicholls SJ, Tuzcu EM, Wolski MPH, Bayturan O, Lavoie A, Uno K (2011). Lowering the triglyceride/high-density lipopreotein cholesterol ratio is associated with the benefical impact of pioglitazone on progression of coronary athersclerosis in diabetic patients. J Am Coll Cardiol..

[39] Vega GL, Barlow CE, Grundy SM, Leonard D, DeFina LF (2014). Triglyceride-to-high-density-lipoprotein-cholesterol ratio is an index of herat disease mortality and of incidence of type 2 diabetes mellitus in men. J Investig Med..

[40] Kulanuwat S, Phonrat B, Tungtrongchitr A, Limwongse C, Chongviriyaphan N, Tungtrongchitr R (2014). Effects of PCSK1 genetic variants on obesity among Thai children and their family members: in relation to health risk, and biochemical and anthropometric parameters. Southeast Asian J Trop Med Public Health..

